# Making a Sustainable Difference to People, Processes and Systems: Whole-Systems Approaches to Process Improvement in Health Systems

**DOI:** 10.3390/ijerph20075232

**Published:** 2023-03-23

**Authors:** Martin McNamara, Marie Ward, Seán Paul Teeling

**Affiliations:** 1 UCD Centre for Interdisciplinary Research, Education & Innovation in Health Systems, UCD School of Nursing, Midwifery & Health Systems, UCD Health Sciences Centre, University College Dublin, Belfield, D04 V1W8 Dublin, Ireland; sean.p.teeling@ucd.ie; 2 Centre for Innovative Human Systems, School of Psychology, Trinity College, The University of Dublin, D02 PN40 Dublin, Ireland; marie.ward@tcd.ie; 3 Centre for Person-Centred Practice Research, Division of Nursing, School of Health Sciences, Queen Margaret University, Musselburgh EH21 6UU, UK

## 1. Introduction

The eighteen papers in this Special Issue, ‘Whole-Systems Approaches to Process Improvement in Health Systems’, address an enduring challenge in healthcare: to improve efficiency with existing or reduced resources, while maintaining safe and effective care. Process improvement methodologies such as Lean, Six Sigma and Lean Six Sigma are increasingly being deployed to address inefficiencies in healthcare. However, a systems perspective is now considered to be the key to sustainable healthcare improvement and results in statistically significant improvement of both patient and service outcomes [1]. It is important, therefore, to pay close attention to the impact of the wider healthcare system on the design and implementation of process improvement methodologies. In the wake of the COVID-19 pandemic, it is clearer than ever that person-centred approaches to change are essential if we are to improve staff and patient experiences. Such approaches help staff to find joy and meaning in their work and to remain working in healthcare, enabling patients to be cared for in an environment that supports their wellbeing in a genuinely holistic sense [2].

This Special Issue focuses on how and to what extent process improvement initiatives across a range of clinical contexts can enhance staff and patient experiences of providing and receiving care and clinical outcomes. These eighteen papers fall into three broad areas:

(1) Lean, Six Sigma and Lean Six Sigma studies embedded within a system-wide improvement programme.
(2) Person-centredness and system improvement.
(3) Systems approaches to change and improvement.

## 2. Lean, Six Sigma and Lean Six Sigma Improvement Studies

Eight out of the nine papers in this part of the Special Issue report on distinct process improvement projects in a single organization [3,4,5,6,7,8,9,10]. Each of these papers outlines how the project achieved its process improvement goals, while taking a person-centred and systems perspective. The key lessons from these projects are considered in a case study on whole-system change that is discussed below [11]. The quality and patient safety improvements reported in the papers include for example: using Lean and person-centred approaches to support the resumption of routine hospital activity following restrictions during the first wave of the COVID-19 pandemic [3]; a reduction of the length of stay for surgeries, leading to fewer healthcare-associated infections [4]; giving nursing and healthcare assistants time to care for patients [6]; an increase in capacity to deliver Basic Life Support across an organisation [8]; surgical notes transferred to electronic platforms to improve legibility and accessibility [9]. The other paper seeks to understand waste in the flow of care for patients in a referral emergency unit using process mapping and value stream analysis of patients in different pathways [12].

## 3. Person-Centredness and System Improvement

Four papers in the Special Issue broach new frontiers in person-centred research [13,14,15,16]. Policy and strategy development globally stress the value of person-centredness as the preferred approach in health and social care [17]. Using structural equation modelling, the first paper examines the statistical significance of the underlying relationships among constructs used in the Person-centred Practice Framework [18], an internationally recognised and globally implemented framework [13]. The results shed light on the importance of multi-disciplinary, shared decision making and working together towards achieving improvement goals. A second paper reports how person-centred methods were used in a realist evaluation to adjudicate context–mechanism–outcome (CMO) configurations [14]. This paper demonstrates the distinctive contribution that person-centred methods can make to realist evaluation, while respecting its underpinning principles and maintaining rigor.

As a realist inquiry, the third paper [15] explores the often-neglected contribution of Lean Six Sigma to person-centred care and cultures. The authors argue that a lack of fidelity to Lean Six Sigma’s philosophical roots creates a divergence between person-centred approaches to transforming care experiences and services, on the one hand, and system-wide quality improvement methods focused on efficiency and clinical outcomes, on the other. The findings demonstrate that Lean Six Sigma approaches may fail to contribute to person-centred cultures when Lean Six Sigma’s original purpose and underpinning philosophy are neglected, and the focus is solely on process standardisation and efficiency gains. The final paper in this section is a realist review of quality care process metrics implementation in nursing and midwifery practices [16]. Quality metrics in healthcare promote standardised care, ensuring consistently high-quality, safe care. The review also demonstrates that combining person-centredness with a systems perspective maximises the likelihood of the successful implementation of improvement efforts.

## 4. Systems Approaches to Change and Improvement

The contested nature of the concept of whole-system change and the lack of agreement in the literature on what it comprises were some the motivations of this Special Issue. Five papers in this final part each use different methods to address system change [19,20,21,22,23]. The first paper [19] looks at discrete-event simulation (DES) modelling, a computer-based operation research technique that models different systems as networks of queues and activities in order to assess, predict, and optimise a proposed or existing system, where changes occur at discrete epochs over time. The authors found that the popularity of DES in healthcare is increasing notably, particularly in emergency departments, where short lead times and the efficient use of resources are essential. In order for DES to be more effective in healthcare, the authors propose three areas of improvement: integration with other process improvement methodologies such as Lean Six Sigma, proper and correct formulation and the incorporation of the behaviour of healthcare staff in order to understand and address cultural obstacles.

In the second paper [20], the authors demonstrate the potential of Implementation Science (IS) laboratories (IS labs) to inform the development of learning health systems that integrate science and practice and overcome the sectoral silos that often constrain whole-system improvements. They show that shared governance and accountability structures, the engagement of stakeholders within and among sectors and genuinely collaborative partnerships enable the integrated, adaptive approach that characterises whole-system improvement. The authors conclude that IS labs offer researchers and practitioners significant opportunities to understand how and why implementation works—or does not work—in specific contexts to sustain innovation.

The aim of the third paper [21] was to better understand the challenges healthcare organisations face when they are striving to improve the quality of their systems and how they overcome them in practice. Drawing on the QUASER (Quality and Safety in Europe by Research) framework, eight case organisations that improved their performance were retrospectively analysed to determine whether, to what extent and how they addressed eight key challenges relating to leadership, culture, politics, structure, emotions, the external environment, education and physical and technological systems. The authors recommend further research into good and/or outstanding organisations to provide insights into how best to achieve a sustainable systems approach to improving healthcare services.

The final two papers adopt a socio-technical systems perspective. The penultimate Access Risk Knowledge (ARK) paper proposes an approach to the management of risk and change through a knowledge engineering system that combines elements of socio-technical system analysis, systems engineering and a mindful governance model [22]. The authors discuss a case study on COVID-19 Infection Prevention and Control. They argue that approaches that support a shared understanding of the system and a common knowledge base not only enhance competence and know-how, but also improve the governance of evidence-based best practice. They conclude that this has the potential to accelerate the embedding of successful organisational change.

The final paper is a meta-analysis case study of eight improvement projects discussed above [11]. The authors reflect on their roles as agents within the system and argue that whole-system change was achieved by acknowledging the organisation’s culture, aligning complex system functionality requirements, activating these requirements to deliver concrete outcomes, developing a shared understanding of future goals and embedding a person-centred approach to whole-system improvement. Through the growing organisation-wide knowledge of the Lean Six Sigma approach and methods underpinned by person-centredness, the organisation is creating a robust and resilient network of those who, in Stigter’s and Cooper’s terms, “can”, “know” and “want” to continuously strive for whole-system improvement [23].

## 5. Conclusions

The eighteen papers in this Special Issue demonstrate how combining person-centredness with a systems perspective maximises the likelihood of the successful implementation of sustainable improvement projects. A narrow focus on tools and techniques alone means that insufficient attention is paid to systemic factors, such as organizational and team relationships, shared purpose and sense making, values that are enacted as well as espoused and organizational capacity. Embedding Lean Six Sigma improvement projects within conceptual frameworks that emphasise systems thinking and person-centredness ensures fidelity to the central principle of respect for the persons on which Lean was founded [24]. It also helps to overcome the pervasive lack of appreciation of how our experiences of ourselves, others and our organizations are shaped by the structure and processes of the systems in which we find ourselves, a phenomenon that Oshry [25] refers to as system blindness. The Special Issue shows how the damaging consequences of system blindness can be overcome by systems thinking and person-centredness, maximizing the potential contribution to the system of employees at all levels in the organization, and of external stakeholders, including patients and their families [15]. Taken together, the papers in the Special Issue demonstrate that, for sustainable whole-system change, it is important to recognise the underlying values and systems dynamics that, if left unacknowledged and unaddressed, could thwart creativity and innovation, impair productive partnerships and undermine improvement programmes.

## References

[1] Komashie A., Ward J., Bashford T., Dickerson T., Kaya G.K., Liu Y., Kuhn I., Günay A., Kohler K., Boddy N. (2021). Systems Approach to Health Service Design, Delivery and Improvement: A Systematic Review and Meta-analysis. BMJ Open.

[2] Lucian Leape Institute (2013). Through the Eyes of the Workforce: Creating Joy, Meaning, and Safer Health Care.

[3] Daly A., Teeling S.P., Garvey S., Ward M., McNamara M. (2022). Using a Combined Lean and Person-Centred Approach to Support the Resumption of Routine Hospital Activity Following the First Wave of COVID-19. Int. J. Environ. Res. Public Health.

[4] Moffatt S., Garry C., McCann H., Teeling S.P., Ward M., McNamara M. (2022). The Use of Lean Six Sigma Methodology in the Reduction of Patient Length of Stay Following Anterior Cruciate Ligament Reconstruction Surgery. Int. J. Environ. Res. Public Health.

[5] Wolfe N., Teeling S.P., Ward M., McNamara M., Koshy L. (2021). Operation Note Transformation: The Application of Lean Six Sigma to Improve the Process of Documenting the Operation Note in a Private Hospital Setting. Int. J. Environ. Res. Public Health.

[6] Egan P., Pierce A., Flynn A., Teeling S.P., Ward M., McNamara M. (2021). Releasing Operating Room Nursing Time to Care Through the Reduction of Surgical Case Preparation Time: A Lean Six Sigma Pilot Study. Int. J. Environ. Res. Public Health.

[7] Daly A., Wolfe N., Teeling S.P., Ward M., McNamara M. (2021). Redesigning the Process for Scheduling Elective Orthopaedic Surgery: A Combined Lean Six Sigma and Person-Centred Approach. Int. J. Environ. Res. Public Health.

[8] Dempsey A., Robinson C., Moffatt N., Hennessy T., Bradshaw A., Teeling S.P., Ward M., McNamara M. (2021). Lean Six Sigma Redesign of a Process for Healthcare Mandatory Education in Basic Life Support—A Pilot Study. Int. J. Environ. Res. Public Health.

[9] Daly A., Teeling S.P., Ward M., McNamara M., Robinson C. (2021). The Use of Lean Six Sigma for Improving Availability of and Access to Emergency Department Data to Facilitate Patient Flow. Int. J. Environ. Res. Public Health.

[10] O’Mahony L., McCarthy K., O’Donoghue J., Teeling S.P., Ward M., McNamara M. (2021). Using Lean Six Sigma to Redesign the Supply Chain to the Operating Room Department of a Private Hospital to Reduce Associated Costs and Release Nursing Time to Care. Int. J. Environ. Res. Public Health.

[11] Ward M.E., Daly A., McNamara M., Garvey S., Teeling S.P. (2022). A Case Study of a Whole System Approach to Improvement in an Acute Hospital Setting. Int. J. Environ. Res. Public Health.

[12] de Barros L.B., Caldas L.P., Bohomol E., Sarantopoulos A., Minatogawa V., Gasparino R.C. (2022). Evaluation of Waste Related to the Admission Process of Low-Complexity Patients in Emergency Services, in Light of the Lean Healthcare Philosophy. Int. J. Environ. Res. Public Health.

[13] McCance T., McCormack B., Slater P., McConnell D. (2021). Examining the Theoretical Relationship Between Constructs in the Person-Centred Practice Framework: A Structural Equation Model. Int. J. Environ. Res. Public Health.

[14] Teeling S.P., Dewing J., Baldie D. (2022). Developing New Methods for Person-Centred Approaches to Adjudicate Context–Mechanism–Outcome Configurations in Realist Evaluation. Int. J. Environ. Res. Public Health.

[15] Teeling S.P., Dewing J., Baldie D. (2021). A Realist Inquiry to Identify the Contribution of Lean Six Sigma to Person-Centred Care and Cultures. Int. J. Environ. Res. Public Health.

[16] Teeling S.P., Davies C., Barnard M., O’Connor L., Coffey A., Lambert V., McNamara M., Tuohy D., Frawley T., Redmond C. (2021). A Rapid Realist Review of Quality Care Process Metrics Implementation in Nursing and Midwifery Practice. Int. J. Environ. Res. Public Health.

[17] World Health Organization (2015). Global Strategy on People-Centred and Integrated Health Services.

[18] McCance T., McCormack B., McCormack B., McCance T., Martin S., McMillan A., Bulley C. (2021). The Person-Centred Practice Framework.

[19] Vázquez-Serrano J.I., Peimbert-García R.E., Cárdenas-Barrón L.E. (2021). Discrete-Event Simulation Modeling in Healthcare: A Comprehensive Review. Int. J. Environ. Res. Public Health.

[20] Flynn R., Brooks S.P., Thomson D., Zimmermann G.L., Johnson D., Wasylak T. (2021). An Implementation Science Laboratory as One Approach to Whole System Improvement: A Canadian Healthcare Perspective. Int. J. Environ. Res. Public Health.

[21] Williams S.J., Best S. (2022). What Does a Systems Approach to Quality Improvement Look Like in Practice?. Int. J. Environ. Res. Public Health.

[22] McDonald N., McKenna L., Vining R., Doyle B., Liang J., Ward M.E., Ulfvengren P., Geary U., Guilfoyle J., Shuhaiber A. (2021). Evaluation of an Access-Risk-Knowledge (ARK) Platform for Governance of Risk and Change in Complex Socio-technical Systems. Int. J. Environ. Res. Public Health.

[23] Stigter M., Cooper C. (2015). Solving the Strategy Delusion: Mobilizing People and Realizing Distinctive Strategies.

[24] Suárez-Barraza M.F., Ramis-Pujol J., Kerbache L. (2011). Thoughts on Kaizen and Its Evolution. Int. J. Lean Six Sigma.

[25] Oshry B. (2018). Context, Context, Context: How Our Blindness to Context Cripples Even the Smartest Organizations.

